# Comparative genomic analysis of 1047 completely sequenced cDNAs from an Arabidopsis-related model halophyte, *Thellungiella halophila*

**DOI:** 10.1186/1471-2229-10-261

**Published:** 2010-11-24

**Authors:** Teruaki Taji, Kenji Komatsu, Taku Katori, Yoshikazu Kawasaki, Yoichi Sakata, Shigeo Tanaka, Masatomo Kobayashi, Atsushi Toyoda, Motoaki Seki, Kazuo Shinozaki

**Affiliations:** 1 Faculty of Applied Bioscience, Tokyo University of Agriculture, 1-1-1 Sakuragaoka, Setagaya-ku, Tokyo 156-8502, Japan; 2 Kihara Institute for Biological Research, Yokohama City University, Maioka 641-12, Totsuka, Yokohama 244-0813, Japan; 3 Experimental Plant Division, BioResource Center, RIKEN Tsukuba Institute, 3-1-1 Koyadai, Tsukuba, Ibaraki 305-0074, Japan; 4 RIKEN Genomic Sciences Center,1-7-22 Suehiro-cho, Tsurumi-ku, Yokohama, 230-0045, Japan; 5 Current address: Comparative Genomics Laboratory, National Institute of Genetics, Yata 1111, Mishima, Shizuoka 411-8540, JAPAN; 6 RIKEN Plant Science Center, 1-7-22 Suehiro-cho, Tsurumi-ku, Yokohama, Kanagawa 230-0045, Japan

## Abstract

**Background:**

*Thellungiella halophila *(also known as *T. salsuginea*) is a model halophyte with a small size, short life cycle, and small genome. *Thellungiella *genes exhibit a high degree of sequence identity with Arabidopsis genes (90% at the cDNA level). We previously generated a full-length enriched cDNA library of *T. halophila *from various tissues and from whole plants treated with salinity, chilling, freezing stress, or ABA. We determined the DNA sequences of 20 000 cDNAs at both the 5'- and 3' ends, and identified 9569 distinct genes.

**Results:**

Here, we completely sequenced 1047 *Thellungiella *full-length cDNAs representing abiotic-stress-related genes, transcription factor genes, and protein phosphatase 2C genes. The predicted coding sequences, 5'-UTRs, and 3'-UTRs were compared with those of orthologous genes from Arabidopsis for length, sequence similarity, and structure. The 5'-UTR sequences of *Thellungiella *and Arabidopsis orthologs shared a significant level of similarity, although the motifs were rearranged. While examining the stress-related *Thellungiella *coding sequences, we found a short splicing variant of *T. halophila salt overly sensitive 1 *(*ThSOS1*), designated *ThSOS1S*. ThSOS1S contains the transmembrane domain of ThSOS1 but lacks the C-terminal hydrophilic region. The expression level of *ThSOS1S *under normal growth conditions was higher than that of *ThSOS1*. We also compared the expression levels of Na^+^-transport-system genes between *Thellungiella *and Arabidopsis by using full-length cDNAs from each species as probes. Several genes that play essential roles in Na^+ ^excretion, compartmentation, and diffusion (*SOS1*, *SOS2*, *NHX1*, and *HKT1*) were expressed at higher levels in *Thellungiella *than in Arabidopsis.

**Conclusions:**

The full-length cDNA sequences obtained in this study will be essential for the ongoing annotation of the *Thellungiella *genome, especially for further improvement of gene prediction. Moreover, they will enable us to find splicing variants such as *ThSOS1S *(AB562331).

## Background

*Thellungiella halophila *(also known as *T. salsuginea*) is used as a model system for understanding abiotic stress tolerance. It shows tolerance not only to extreme salinity stress, but also to chilling, freezing, and ozone stresses [1-10]. *Thellungiella *is closely related to Arabidopsis, with 90% cDNA sequence identity between the two species, and it can be easily transformed by using the floral dipping method [1,11]. *Thellungiella *has a number of other features useful for genetic research, such as small size, short life cycle, high seed number, and self-compatibility.

The Arabidopsis genome sequence and other genetic resources, including collections of full-length cDNAs, have provided powerful tools for comparative genomics to understand the biology and evolution of other plants [3,5,12]. In particular, highly accurate full-length cDNA sequences that span the entire protein-coding region of a given gene can advance comparative, functional, and structural genome analyses. The accurate prediction of protein-coding regions in genome sequences is limited by the difficulty of finding islands of coding sequences within an ocean of noncoding DNA, and by the complexity of individual genes that may code for multiple peptides through alternative splicing. The sequence data from full-length cDNAs has contributed to the accuracy of annotation and to improving gene prediction in Arabidopsis [13-15]. For these reasons, we have been working to collect similar data for *Thellungiella*.

We previously reported construction of a full-length cDNA library of *Thellungiella *derived from various tissues and from whole seedlings subjected to environmental stress treatments, including high salinity, chilling, freezing, and abscisic acid (ABA). We obtained a total of 35 171 sequences from 20 000 clones, and named them RIKEN *Thellungiella *Full-length (RTFL) cDNA clones. These sequences were assembled by using the CAP3 method and were clustered into 9569 nonredundant cDNA groups [16].

*Thellungiella *has an effective system for suppressing Na^+ ^influx and for excreting Na^+ ^[3]. It also exhibits high potassium/sodium selectivity, according to electrophysiological analysis of instantaneous current [4]. This implies that *Thellungiella *has ion channels with specific features that lead to superior sodium/potassium homeostasis. Membrane transporters have been shown to be important components of salt tolerance mechanisms in other species on account of their regulation of ion homeostasis. For example, the SALT OVERLY SENSITIVE (SOS) pathway is a well-defined pathway in Arabidopsis for the regulation of sodium ion homeostasis during plant growth under salinity stress [17,18]. In this pathway, a calcium-binding protein, SOS3, perceives a change in intracellular calcium concentration induced by salt stress and then binds to and activates SOS2, a serine/threonine protein kinase. The SOS3-SOS2 complex increases the expression and activity of SOS1, which encodes a plasma membrane Na^+^/H^+ ^exchanger (antiporter) [19,20]. Activated SOS1 transports cytosolic sodium out of the cell, reducing the cellular build-up of toxic levels of sodium [17]. The *Thellungiella *SOS1 gene, *ThSOS1*, has a conserved amino acid sequence and protein structure with orthologous genes from Arabidopsis and other plants [21]. Transgenic *Thellungiella *plants in which *ThSOS1 *transcript levels were reduced by RNA interference (RNAi) showed lower salt tolerance than wild-type plants, suggesting that SOS1 is critical for salt tolerance in halophytic species as well as in glycophytic species such as Arabidopsis [21]. Recently, a 193-kb *Thellungiella *BAC clone containing the putative *SOS1 *locus was sequenced, annotated, and compared with the sequence in the orthologous 146-kb region of the Arabidopsis genome on chromosome 2 [22].

Here, we selected 1047 cDNAs for genes related to salt stress, transcription factors, transporters, and protein phosphatase 2Cs from 9569 individual RTFL clones, and determined the complete sequences. We then predicted the coding sequence (CDS), 5'-UTR, and 3'-UTR for each of the cDNAs and compared them with the corresponding regions from the orthologous Arabidopsis genes. We also compared the expression levels of *Thellungiella *and Arabidopsis Na^+^-transport system genes by using full-length cDNAs to probe Northern blots under equal conditions of hybridization and detection.

## Results and Discussion

### Selection and complete sequencing of 1047 full-length cDNAs

**Figure 1:**
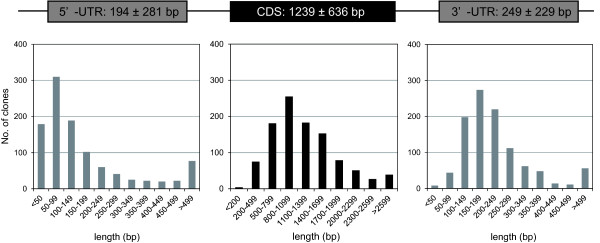
**Size distribution of 5'-untranslated regions (UTRs), coding sequences (CDS), and 3'-UTRs among 1047 full-length cDNAs from *Thellungiella halophila***. The diagram above the graphs shows mean lengths (±standard deviation) of each region.

We selected 1047 cDNA clones representing salt-stress-related genes, transcription factors, and protein phosphatase 2Cs for full-insert sequencing (Table 1). The 1047 cDNA clones were completely sequenced, and the CDS, 5'-UTR, and 3'-UTR were predicted for each (see Additional file 1, Table S1 and Additional file 2, Table S2). The distribution of 5'-UTR, CDS, and 3'-UTR lengths is illustrated in Figure 1; their average lengths were 206 ± 295 bp, 1214 ± 650 bp, and 270 ± 283 bp, respectively. These results are comparable to the results obtained for CAP-trapper full-length cDNA collections from other plant species, including Arabidopsis (5'-UTR, 149 bp; CDS, 1206 bp; 3'-UTR, 238 bp; TAIR8 dataset of The Arabidopsis Information Resource (TAIR, ftp://ftp.arabidopsis.org/home/tair/Sequences/blast_datasets/TAIR8_blastsets/), maize (5'-UTR, 99 bp; 3'-UTR, 206 bp [23]), rice (5'-UTR, 259 bp; 3'-UTR, 398 bp [24]), and poplar (5'-UTR, 109 bp; 3'-UTR, 228 bp [25]).

**Table 1:** Classification of 1250 full-sequenced cDNAs

classification	number of cDNAs	references
**433 salt stress related genes**	**367**	
*Thellungiella *salt stress inducible genes	127	Taji *et al*., 2004 *Plant Physiol* *Gong et al.*, 2005 *Plant J*. Wong *et al.*, 2005 *Plant Mol Biol*. Taji *et al.*, unpublished data
salt stress inducible genes in *Thellungiella *and Arabidopsis	191	
constitutive high-expressed genes in *Thellungiella *compared with Arabidopsis	78	
genes encoding Na^+ ^transporter	9	
overlapping genes in EST libraries of abiotic stressed plants from Thellungiella	30	
**response to abiotic or biotic stress classified by Gene Ontology^a^**	**450**	Taji *et al*., 2009 *BMC Plant Biol*.
		
**transcription factor classified by Gene Ontology^a^**	**285**	Taji *et al*., 2009 *BMC Plant Biol*.
		
**protein phosphatase 2C**	**24**	Taji *et al*., 2009 *BMC Plant Biol*.

total	1126	

**total after elimination of overlapped cDNAs**	**1047**	

^a ^Gene Ontlogy (GO) terms were obtained using InterPro. According to the GO terms, RTFL clones were classified by using Plant GO Slim http://www.geneontology.org/GO.slims.shtml

### Comparison of CDS, 5'-UTR, and 3'-UTR sequences between orthologous genes *in *Thellungiella and Arabidopsis

**Figure 2:**
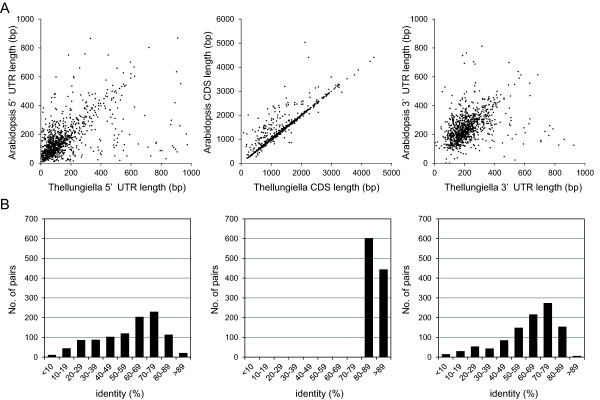
**Comparison of the length and % identity of 5'-UTRs, CDSs, and 3'-UTRs between *Thellungiella *cDNAs and the orthologous genes in Arabidopsis**. (A) Comparison of the lengths of 5'-UTRs (left), CDSs (center), and 3'-UTRs (right) between *Thellungiella *cDNAs and the orthologous genes from Arabidopsis. (B) Nucleotide sequence identity of 5'-UTRs (left), CDSs (center), and 3'-UTRs (right) between *Thellungiella *cDNAs and the orthologous genes from Arabidopsis.

To assess the quality of the completely sequenced cDNAs, we performed BLAST analysis using CDS sequences against nucleotide or peptide sequences from the TAIR8 dataset (see Additional file 1, Table S1 and Additional file 2, Table S2) and identified Arabidopsis orthologs of the 1047 *Thellungiella *genes. The average lengths of the Arabidopsis orthologous CDSs, 5'-UTRs, and 3'-UTRs were 1331 ± 698 bp, 160 ± 145 bp, and 241 ± 146 bp, respectively. Figure 2 compares lengths and identities between the CDS, 5'-UTR, and 3'-UTR regions of the 1047 *Thellungiella *cDNAs and those of the orthologous genes from Arabidopsis. Most CDS pairs showed highly similar lengths, whereas the 5'- and 3'-UTR pairs showed significant variation in length (Figure 2A). The average nucleotide identity within homologous CDS pairs was 87%, whereas the average identity within the 5'- and 3'-UTR pairs was only 57% to 61% (Figure 2B). A previous analysis of the transcriptional differences between *Thellungiella *and Arabidopsis showed that Arabidopsis has a global defense strategy that requires bulk gene expression, while *Thellungiella *induces expression of genes functioning in protein folding, posttranslational modification, and protein redistribution [5]. The sequence diversity in the 5'- and 3'-UTR pairs may be involved in the posttranslational regulation of stress tolerance mechanisms in *Thellungiella*.

### Comparison of structure of UTR regions between Thellungiella and Arabidopsis

**Figure 3:**
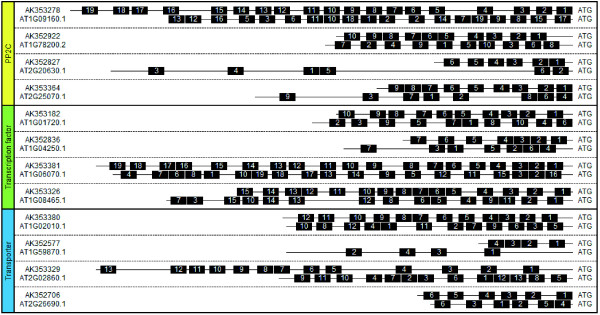
**Arrangement of motifs identified in 5'-UTR regions of nine orthologous gene pairs in *Thellungiella *and Arabidopsis**. The motif families shared in 5'-UTRs of nine randomly selected *Thellungiella *and Arabidopsis orthologous pairs from PP2Cs, transcription factors and transporters were identified using Dragon Motif Builder [26]. Black boxes indicate motifs. Identical numbers within each pair indicate identical motifs. Motif sequences are shown in Additional file 4, Table S3.

To compare the overall architecture of the UTRs between *Thellungiella *and Arabidopsis, we randomly selected 10 orthologous pairs with 5'-UTRs of least 50 bp in length from PP2Cs, transcription factors and transporters, respectively. We identified motif families shared between the 5'-UTRs of *Thellungiella *and Arabidopsis orthologs using the Dragon Motif Builder system [26]. Analyzing each of the orthologous pairs individually, we compared the order of the shared motifs between each pair (Additional file 3, Figure S1). Figure 3 shows the arrangement of the motifs in 5'-UTR regions in nine orthologous gene pairs in *Thellungiella *and Arabidopsis. These motif sequences are shown in Additional file 4, Table S3. The members of each orthologous 5'-UTR pair shared 4 to 19 motifs; however, positional rearrangements were found between the members of each pair. Similar positional rearrangements of 5'-UTR motifs were reported in a comparison of 48 pairs of orthologous sequences between common carp and zebrafish [27]. The presence of such shared motif families suggests the existence of regulatory components common to both species.

### Structural comparison of ThSOS1 and splicing variant ThSOS1S

**Figure 4:**
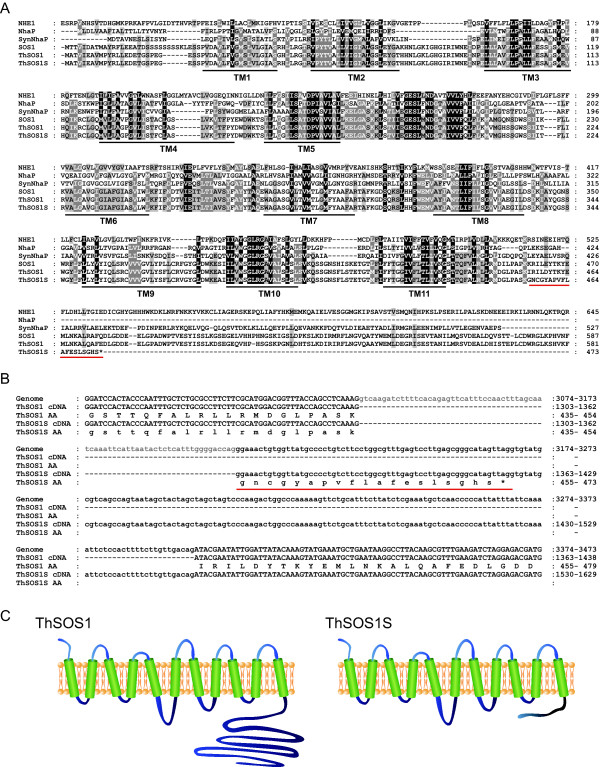
**Comparison of the structures of ThSOS1 and ThSOS1S**. (A) Alignment of the transmembrane regions of NHE1 (P19634), SynNhaP (D90910), SOS1 (AF256224), and ThSOS1 (EF207775.1) and the complete sequences of NhaP (P13738) and ThSOS1S constructed with ClustalW ver. 1.83. Amino acid residues conserved in all sequences are highlighted in black, and conserved substitutions are shown in gray. Predicted membrane spanning regions (TM) were determined by TMpred [42] and are marked under the alignment. (B) Close-up alignment of the nucleotide sequences of *ThSOS1*, *ThSOS1S*, and the genome with the deduced amino acid sequences from Gly-435 to Asp-479. The nucleotides conserved in *ThSOS1 *and *ThSOS1S *are written in capital letters, and the *ThSOS1S*-specific sequence is written in lower-case letters. Intron sequence is shown in gray letters. The predicted amino acid sequence of ThSOS1 is shown in capital letters, and that of ThSOS1S in lower-case letters. The 19aa insert of ThSOS1S is red-underlined. The asterisk shows the point at which the ThSOS1S protein is truncated. (C) Hypothetical secondary structure models of ThSOS1 and ThSOS1S.

Only one clone was orthologous to Arabidopsis SOS1 among 20 000 sequenced *Thellungiella *cDNAs [16]. The deduced protein was a splice variant of ThSOS1 (Acc. No. EF207775.1). In the variant, an exon encoding 19 amino acid (aa) residues (60 nucleotides) followed by a stop codon was inserted at the beginning of the 15th exon of *ThSOS1 *(Figure 4A, B). We named the short variant *ThSOS1S*, for *Thellungiella halophila *Salt Overly Sensitive 1 Short form. ThSOS1 comprises an N-terminal, integral membrane domain (responsible for Na^+ ^transport) and a C-terminal hydrophilic region. In contrast, the predicted ThSOS1S protein has the transmembrane domain of ThSOS1 but lacks the C-terminal hydrophilic region, because the stop codon occurs just after the sequence encoding the transmembrane domain (Figure 4C).

The transmembrane portion of ThSOS1/ThSOS1S has sequence similarities with plasma membrane Na^+^/H^+ ^exchangers of animal, bacterial, and fungal cells [20]. In animal cells, Na^+^/H^+ ^exchanger 1 (NHE1) functions as a Na^+^/H^+ ^antiporter to maintain pH homeostasis [28]. NHE1 has a C-terminal tail of ~300 aa, which is important in regulating the Na^+^/H^+ ^antiporter activity through phosphorylation or binding of regulatory proteins [29]. The *Synechocystis *Na^+^/H^+ ^antiporter SynNhaP also has a long hydrophilic C-terminal tail (100 aa). In SynNhaP, the deletion of a 56-aa hydrophilic terminal region partially inhibited the antiporter activity, and replacement of the long C-terminal tail with the orthologous region from the halotolerant cyanobacterium *Aphanothece halophytica*, ApNhaP, altered its ion specificity [30]. Arabidopsis Na^+^/H^+ ^antiporter SOS1 has 12 predicted transmembrane domains in the N-terminal region and a long cytoplasmic tail of ~700 aa at the C-terminus [20]. The predicted cytoplasmic tail of SOS1 interacts with radical-induced cell death 1 (RCD1), a regulator of oxidative stress responses under salt or oxidative stress. Like *rcd1 *mutants, *sos1 *mutants show an altered sensitivity to oxidative stresses [31]. These results suggest that the long C-terminal tail mediates not only the regulation of transport activity with a variety of intracellular regulatory proteins, but also the ion specificity and the cross-talk with other stress tolerance mechanisms.

The N-terminal transmembrane region of SOS1 shows high similarity among various organisms (Figure 4A), whereas there is no significant similarity among the C-terminal regions [30]. The C-terminal sequence variation may result in different functions for this region among different organisms. In particular, NhaP, a Na^+^/H^+ ^antiporter of *Pseudomonas aeruginosa*, is highly homologous to SOS1, NHE1, SynNhaP, and ApNha1 (Figure 4A), but it does not have the C-terminal long tail [32]. ThSOS1S is similar to NhaP in that it contains only a Na^+^/H^+^-exchanger domain in the transmembrane domain. It is possible that ThSOS1S functions as an Na^+^/H^+ ^antiporter whereas ThSOS1 functions not only in salt stress response (via the N-terminal Na^+^/H^+ ^antiporter), but also in response to other abiotic stresses (via the long C-terminal tail).

### Expression levels of ThSOS1 and ThSOS1S

**Figure 5:**
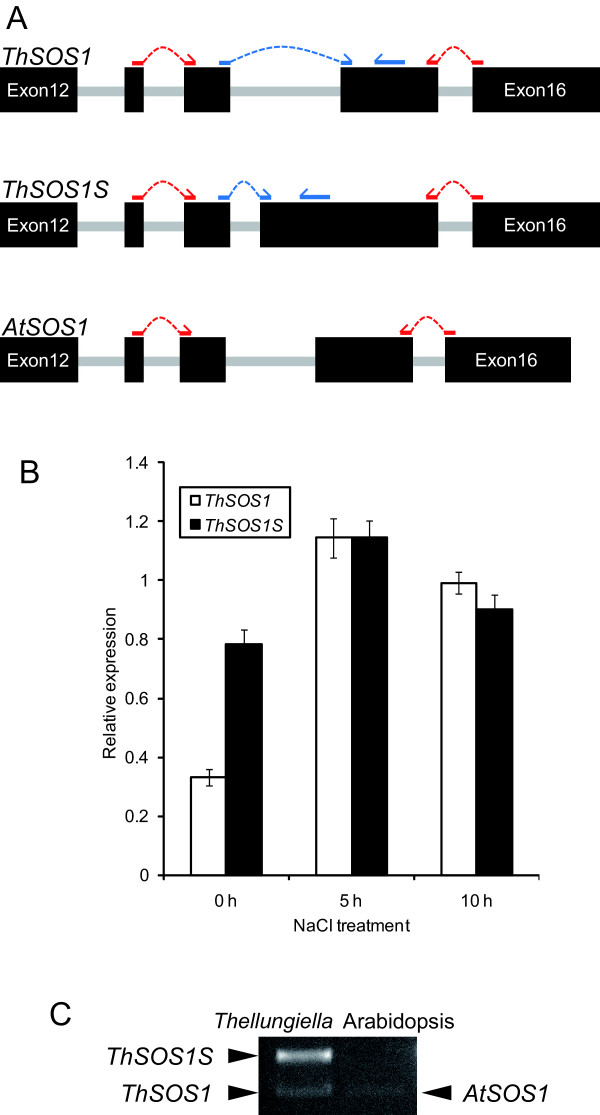
**Effect of salt stress on transcript levels of *ThSOS1 *and *ThSOS1S***. (A) Schematic of the corresponding regions of the primers in *ThSOS1*, *ThSOS1S *and *AtSOS1 *for RT-PCR analysis. Exons are indicated as filled boxes and introns as spaces between them. (B) Relative expression of *ThSOS1 *and *ThSOS1S *under salt stress. Two-week-old *Thellungiella *seedlings were exposed to 250 mM NaCl stress for 0, 5, or 10 h. Total RNA was reversed-transcribed into cDNA and used as a template for quantitative RT-PCR. The qRT-PCR performed using blue-primer sets in (A). Relative transcript levels were normalized to *Actin2 *mRNA. Data are the mean ± SD for three individual experiments (*n *= 3). (C) Semiquantitative gene expression of *SOS1 *splice variants in *Thellungiella *and Arabidopsis under normal growth conditions. The RT-PCR performed using red-primer sets in (A), which were able to detect these splice variants.

We performed qRT-PCR analysis of *ThSOS1 *and *ThSOS1S *expression by using primers specific to each of these splice variants (Figure 5A). We detected both transcripts, suggesting that *Thellungiella *normally produces both forms (Figure 5B). Interestingly, the expression level of *ThSOS1S *under normal growth conditions was higher than that of *ThSOS1*. The expression level of *SOS1 *in *Thellungiella *is higher than that in Arabidopsis when full-length cDNAs are used as probes, especially under normal growth conditions [3]. These data suggest that the high expression of *ThSOS1 *detected under normal growth conditions derive from the high expression level of *ThSOS1S*. To confirm the existence of such a similar splice variant in Arabidopsis, RT-PCR was performed using primer sets that are able to detect the splice variants in *Thellungiella *and Arabidopsis. The short splice variant corresponding to *ThSOS1S *was not detected in Arabidopsis, whereas both splice variants were detected in *Thellungiella *(Figure 5C). This result suggests that the short splice variant of *SOS1 *is specific to *Thellungiella*.

### Expression profiles of Na^+ ^transport genes of Thellungiella and Arabidopsis

**Figure 6:**
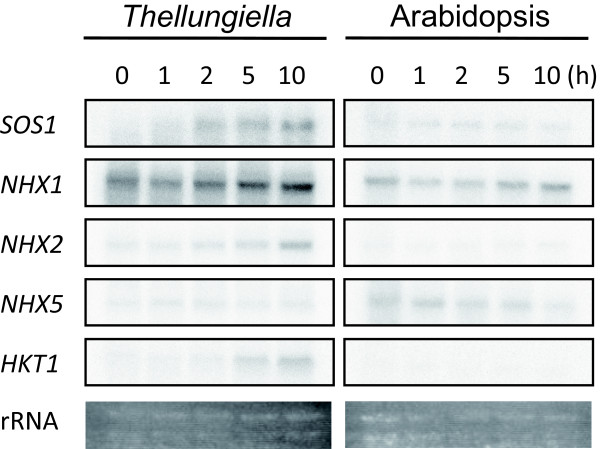
**RNA gel blot analysis of Na^+ ^transport system genes in *Thellungiella *and Arabidopsis**. Total RNA was prepared from 3-week-old *Thellungiella *and Arabidopsis plants subjected to 250 mM NaCl stress treatment for the periods indicated. Each lane was loaded with 10 μg total RNA. The probes were the following full-length cDNA fragments from *Thellungiella *and Arabidopsis, respectively: *SOS1 *(AK353556), *NHX1 *(AK352788), *NHX2 *(AK353509), *NHX5 *(AK353024), and *HKT1 *(AK353477).

The set of completely sequenced *Thellungiella *cDNA clones contains several genes that function in the Na^+ ^transport system, including *SOS1*, *NHX1*, *NHX2*, *NHX5*, and high affinity K^+ ^transporter 1 (*HKT1*). We performed RNA blot analysis of these genes in both *Thellungiella *and Arabidopsis using full-length cDNAs as probes. In each case, RNA blots of a given species were hybridized with probes derived from that same species, with conditions of probe radioactivity, hybridization, and exposure period normalized between the two species. The expression levels of *SOS1*, *NHX1*, *NHX2 *and *HKT1 *in *Thellungiella *were higher than those in Arabidopsis under both normal and high-salinity conditions (Figure 6). SOS1, NHX1, and HKT1 play essential roles in salt tolerance in Arabidopsis [33-35], and transgenic plants overexpressing either *SOS1 *or *NHX1 *show higher tolerance to salt stress than do wild-type plants [18,36]. In particular, the expression level of *NHX1 *was very high in *Thellungiella *under both normal- and high-salinity conditions, suggesting that the constitutively high expression of molecules functioning in Na^+ ^transport may partly account for the high salinity tolerance of *Thellungiella*.

It is difficult to understand the relationship between *HKT1 *expression and salinity tolerance. On the one hand, the overexpression of *AtHKT1 *increased sensitivity to NaCl (but not to LiCl or KCl) compared with wild-type plants [37]. On the other hand, the ectopic production of wheat HKT1 containing specific amino acid substitutions (A240V, Q270L, or N365S) enhanced NaCl tolerance in yeast [38]. We compared the deduced amino acid sequences of HKT1 among Arabidopsis, *Thellungiella*, and wheat to search for point mutations inducing higher salt tolerance in *Thellungiella *HKT1. However, none of the mutations that gave high salt tolerance in the wheat HKT1 experiment was found in *Thellungiella *HKT1 (data not shown). It remains unknown whether other differences in the HKT1 sequences between *Thellungiella *and Arabidopsis confer salinity tolerance to *Thellungiella*. Arabidopsis HKT1 transports only Na^+^, not K^+^, because in the K^+ ^channel motif GYG, which is critical for K^+ ^selectivity, the first glycine is replaced with serine (Ser-68) [39]. The same position in the *Thellungiella *HKT1 ortholog is also serine (Ser-68), suggesting that *Thellungiella *HKT1 also transports only Na^+^.

## Conclusions

We sequenced 1047 *Thellungiella *cDNAs and used this information to compare the responses of *Thellungiella *and Arabidopsis to high-salinity conditions. The full-length cDNA sequences will contribute to annotation of the *Thellungiella *genome and will improve gene predictions. Moreover, these fully sequenced cDNAs will enable finding splicing variants such as *ThSOS1S*. RNA blot analysis indicated that the extreme salt tolerance of *Thellungiella *might be attributable to the constitutively higher expression of genes functioning in the Na^+ ^transport system.

### Data access

Sequences from this study have been deposited in NCBI GenBank under accession numbers [GenBank: AK352512] to [GenBank: AK353558]. The RTFL clones are available for distribution from the RIKEN Bioresource Center http://www.brc.riken.go.jp/lab/epd/Eng/.

## Methods

### Determination of CDSs, 5'-UTRs, and 3'-UTRs of full-length cDNAs

The locations of CDSs were determined with the EMBOSS getorf program (ver. 6 [40]), which identifies the longest stretch of uninterrupted sequence between a start codon (ATG) and stop codon (TGA, TAG, TAA) in the 5'- to 3' direction as the predicted CDS. The sequences before and after each predicted CDS were designated as the 5'- and 3'-UTRs, respectively. The 3' poly(A)-tail lengths were not included when determining the UTR lengths.

### Identification of orthologous genes in Arabidopsis and Thellungiella

The CDS data set of 1047 *Thellungiella *cDNAs was compared with the gene sequences in The Arabidopsis Information Resource (TAIR8) by using BLAST searches (ver. 2.2.17 [41]). The top hit in each BLAST search was assumed to be the Arabidopsis ortholog.

### Plant materials and growth conditions

Seeds of *Thellungiella halophila *(Shang Dong ecotype) and *Arabidopsis thaliana *(Columbia-0 ecotype) were sown on MS agar plates containing full-strength MS, 0.8% (w/v) agar, and 1% sucrose with vitamin mixture (10 mg L^-1 ^myoinositol, 200 μg L^-1 ^glycine, 50 μg L^-1 ^nicotinic acid, 50 μg L^-1 ^pyridoxine hydrochloride, 10 μg L^-1 ^thiamin hydrochloride, pH 5.7) and the plates were sealed with surgical tape. The seeds were stratified at 4°C for 7 days and then transferred to 80 μmol m^-2^s^-1 ^irradiance under an 8/16-h day/night cycle at 22°C for germination and growth.

### Northern analysis

Three-week-old Arabidopsis and *Thellungiella *plants that had been grown on 1/2 MS plates were soaked in 250 mM NaCl solution for 1, 2, 5, or 10 h. Total RNA was extracted by using RNAiso reagent (TaKaRa, Japan). Total RNA (10 μg) was fractionated in 1% agarose gel containing formaldehyde and blotted onto a nylon membrane using 20× SSC. DNA fragments of the full-length cNDAs for *AtSOS1 *(RAFL09-06-M16), *AtSOS2 *(RAFL09-61-G03), *AtNHX1 *(RAFL09-87-C17), *AtNHX2 *(RAFL07-95-G07), *AtNHX5 *(RAFL09-15-L23), *AtHKT1 *(RAFL15-31-F16), *ThSOS1 *(RTFL01-052_J19), *ThSOS2 *(RTFL01-012_C03), *ThNHX1 *(RTFL01-029_F03), *ThNHX2 *(RTFL01-047_K14), *ThNHX5 *(RTFL01-046_D22) and *ThHKT1 *(RTFL01-044_N15) were used as probes. Probes were labeled with [^32^P]dCTP using a DNA Labeling Kit ver. 2 (TaKaRa, Japan), and membranes were hybridized with ^32^P-labeled fragments at 65°C overnight. The membranes were washed 3 times with 1× SSC, 1% SDS for 3 min at room temperature; then once with 1× SSC, 1% SDS for 15 min at room temperature; then twice with 0.1× SSC, 0.1% SDS for 15 min at 65°C.

### Semiquantitative or quantitative RT-PCR

For cDNA synthesis, 1 μg of total RNA was first treated with DNaseI (Sigma-Aldrich, USA) for 15 min at room temperature, and the enzyme was inactivated by heating at 70°C for 10 min. Reverse transcription was performed with the ThermoScript RT-PCR system (Invitrogen, USA) according to the manufacturer's instructions. Synthesized cDNAs were purified using the Gel Extraction kit. Semiquantitative RT-PCR analysis for *ThSOS1 *and *AtSOS1 *expression was performed using 1 μl of the cDNA, primer sets (*ThSOS1*; Exon13-14 forward 5'-CCGAGACAGGAACAATGTTTAT-3' and Exon15-16 reverse 5'-AGTAAGCTGCCTGAACACCAT-3', *AtSOS1*; Exon13-14 forward 5'-AGGAGACTGGAACATTGTTTCT-3' and Exon15-16 reverse 5'-AGTAAGTTGCTTGCACACCATT-3') and BioTaq polymerase with the supplied buffer and dNTP (BIOLINE, UK). The PCR conditions were as follows: 30 cycles of 95°C for 30 s, 55°C for 30 s and 72°C for 30 s. A 10 μl aliquot of each PCR reaction was separated on an agarose gel. The comparative expression analysis of *ThSOS1 *and *ThSOS1S *was performed by quantitative PCR with LightCycler Systems for Real-Time PCR (Roche Applied Science, Japan) using the LightCycler-FastStart DNA Master SYBR Green I kit (Roche Applied Science, Japan) and the primer sets (*ThSOS1*; Exon14-15 forward 5'- CGGTTTACCAGCCTCAAAGATACGAA-3' and reverse 5'- AAACGCTTGTAAGGCCTTATTCAGCAT-3', *ThSOS1S*; Exon14-15 forward 5'- TTTACCAGCCTCAAAGGGAAACTGTG-3' and *ThSOS1S *specific reverse 5'- CACCTAACTATGCCCGCTCAAGGA-3' and *Actin2*; forward 5'- AGTGGTCGTACAACCGGTATTGT-3' and reverse 5'- GATGGCATGAGGAAGAGAGAAAC-3') according to the manufacturer's instructions. The PCR conditions were as follows: 40 cycles of 95°C for 10 s, 55°C for 10 s and 72°C for 10 s. The relative expressions were calculated using the Second Derivative Maximum Method on LightCycler Data Analysis software (Roche Applied Science, Japan). The *Actin2 *(At3g18730) co-orthologous gene was used to normalize *ThSOS1 *and *ThSOS1S *expressions.

### Measurement of plant Na^+ ^content

Two-week-old Arabidopsis and *Thellungiella *plants grown on 1/2 MS agar plates were transferred to plates containing 1/2 MS agar medium plus 250 mM NaCl. Plants were harvested at 1, 3, 5, 7, 10, 14, 21, and 28 days after transfer. For each sample, five plants were pooled and soaked in 5 mL sterile distilled water. The leaf-water mixture was boiled for 15 min, filtered through a 0.2-μm filter (Toyo Roshi Kaisha, Ltd.), and diluted 20-fold. The solution was analyzed by using a Shim-pack IC-C3/C3 (S) column (Shimadzu, Japan) on a Shimadzu PIA-1000 Personal Ion Analyzer (Shimadzu, Japan).

## Authors' contributions

TT contributed to and participated in the entire study and drafted the manuscript. KK performed the bioinformatics analyses (annotation, prediction of CDS, 5'-UTR and 3'-UTR and comparative analysis). TK carried out molecular biology studies and a part of bioinformatics analyses (comparative analysis). KY carried out the Northern analysis. AT conducted sequencing of the cDNA clones and registration in DDBJ. KM planned the study and obtained funding for the research. YS and ST helped draft the manuscript. KS and MS coordinated the project and helped draft the manuscript.

## Supplementary Material

Additional file 1 **Table S1, Title**. Predicted protein coding features, annotation for RIKEN T. halophila full-length cDNA (RTFL) collection. Abbreviations: AA, amino acid, CDS, coding sequence, NA, nucleic acid. Click here for file

Additional file 2 **Table S2, Title**. Predicted 5', 3'-untranslated region of RIKEN T. halophila full-length cDNA (RTFL) collection. Abbreviation: UTR, untranslated region. Click here for file

Additional file 3 **Figure S1, Title**. Arrangement of motifs identified in 5'-UTR regions of 30 orthologous gene pairs in *Thellungiella *and Arabidopsis. Click here for file

Additional file 4 **Table S3, Title**. Motif sequences. Click here for file
